# Cancer Cells in Sleep Mode: Wake Them to Eliminate or Keep Them Asleep Forever?

**DOI:** 10.3390/cells13232022

**Published:** 2024-12-06

**Authors:** Wenjie Liu, Antal H. Kovacs, Jinqiang Hou

**Affiliations:** 1 Department of Chemistry, Lakehead University, 955 Oliver Rd, Thunder Bay, ON P7B 5E1, Canada; wliu28@lakeheadu.ca (W.L.); ahkovacs@lakeheadu.ca (A.H.K.); 2 Thunder Bay Regional Health Research Institute, 980 Oliver Road, Thunder Bay, ON P7B 6V4, Canada

**Keywords:** cancer dormancy, metastasis, tumor microenvironment

## Abstract

Cancer cell dormancy is a critical phase in cancer development, wherein cancer cells exist in a latent state marked by temporary but reversible growth arrest. This dormancy phase contributes to anticancer drug resistance, cancer recurrence, and metastasis. Treatment strategies aimed at cancer dormancy can be categorized into two contradictory approaches: inducing cancer cells into a dormant state or eliminating dormant cells. While the former seeks to establish permanent dormancy, the latter aims at eradicating this small population of dormant cells. In this review, we explore the current advancements in therapeutic methods targeting cancer cell dormancy and discuss future strategies. The concept of cancer cell dormancy has emerged as a promising avenue for novel cancer treatments, holding the potential for breakthroughs in the future.

## 1. Introduction

Dormancy is an adaptive strategy used by various species to cope with unfavorable and challenging conditions, ensuring their survival. Cancer cell dormancy is frequently observed in various cancer types, with many patients having dormant cancer cells for extended periods while remaining clinically cancer-free [1]. Cancer cell dormancy refers to a state where cancer cells remain latent, marked by a temporary but reversible growth arrest [2,3]. This underscores the potential of inducing and maintaining cancer cells in a dormant and harmless state for therapeutic purposes. However, cancer cell dormancy is also a crucial factor contributing to tumor relapse, progression, and drug resistance, as these cells can survive initial chemotherapy and later be reawakened. Therefore, there have been suggestions to target and eliminate dormant cells that survive chemotherapy to prevent cancer recurrence. The concept of targeting cancer cell dormancy presents a promising novel approach in cancer treatment strategies. However, it has sparked debates about whether dormancy should be induced or completely eradicated. Hence, this review article aims to provide insightful perspectives on cancer cell dormancy and explore strategies for developing anticancer therapies that specifically target dormant cancer cells.

## 2. Major Characteristics of Cancer Cell Dormancy

Dormant cancer cell-associated biomarkers include molecules involved in proliferation and apoptosis, such as Ki67 and p27. Typically, dormant cancer cells exhibit elevated levels of the cell cycle inhibitors p21 and p27, along with reduced levels of the proliferation marker Ki67 [4,5]. Recent studies have identified additional markers like NR2F1 and DEC2 that indicate dormancy in disseminated tumor cells (DTCs) [6,7]. Dormant cancer cells also demonstrate reduced expression of the mitogenic pathway (extracellular signal-regulated kinase, ERK) and enhanced activation of the growth arrest pathway (p38 mitogen-activated protein kinase, MAPK). Notably, the p38^High^/ERK^Low^ phenotype serves as a prototype marker for dormant cells [8,9,10]. These dormancy markers could facilitate early monitoring of metastatic relapse. Dormant cancer cells also exhibit other characteristics, such as altered metabolism [11,12], upregulated adaptation to oxidative stress [13], and tighter chromatin compaction [14].

The mechanisms responsible for cancer cell dormancy remain incompletely understood; both intracellular and extracellular regulatory mechanisms play important roles in regulating dormancy. Intracellular factors include those involved in cell growth, such as cell cycle regulation, proliferation and apoptosis, as well as metabolic alterations and autophagy. For instance, transforming growth factor-β2 (TGF-β2) and p38/MAPK signaling pathways promote cell dormancy by downregulating cyclin-dependent kinases (CDKs) and upregulating CDK-inhibitory factors [9,15,16,17]. It has been suggested that autophagy supports dormant cancer cells by maintaining amino acid levels, preventing energy crises, and promoting ATP production [18,19]. Extracellular factors include the extracellular matrix (ECM), environmental stress, and the immune system. For instance, robust evidence supports the idea that certain ECM proteins can promote cell dormancy. One study suggested that type III collagen is a key ECM component necessary for maintaining dormancy [20]. Another study indicated that, in human breast cancer cell lines capable of entering a long-term dormant state, there is consistent production and assembly of a fibronectin matrix through α_5_β_1_ integrin-mediated adhesion and rho-associated kinase (ROCK)-mediated cell tension. The degradation of this fibronectin matrix by matrix metalloproteinase 2 (MMP-2) is necessary for dormant cells to exit dormancy and resume a proliferative state [21]. Cellular dormancy can also be influenced by extracellular stress, such as hypoxia. Hypoxia promotes dormancy by activating the expression of genes related to apoptosis, survival, and the cell cycling of dormant cells [22,23,24,25], while also modulating various dormancy-related pathways, including MAPK [10,25]. Hypoxic conditions have been reported to induce dormancy in HPV-positive cancer [26], colorectal cancer [27], lung cancer [24], breast cancer, and head and neck cancer [6].

## 3. Strategies of Inducing Cancer Cell Dormancy

Promoting and maintaining a dormant state in cancer may offer a treatment option to control tumor growth and prevent metastasis to some extent, which may lead to an improvement in the health span of patients. This strategy will not lead to complete remission in advanced disease but may offer the patient more time before significant recurrence and metastasis occurs. Research regarding this concept is still at an early stage, but several strategies have been developed to maintain and induce dormancy. Activating dormancy-inducing factors or pathways, suppressing proliferation-related factors or pathways, and supplying components of the dormant niche are potential strategies for maintaining dormancy. Additionally, many other strategies of inducing dormancy have been proposed, including anti-angiogenic therapies, immune-mediated therapies, epigenetic modulation, and metabolic interventions.

### 3.1. Activating Dormancy-Inducing Factors or Inhibiting Proliferation-Related Factors

Dormancy can be induced either by increasing or maintaining the expression of dormancy-inducing factors or by inhibiting growth factors. The molecules discussed in this section represent promising therapeutic targets for developing drugs that modulate cancer cell dormancy.

Inhibiting proliferation-related factors such as focal adhesion kinase (FAK) [28], urokinase receptors (uPARs) [28,29], ERK activities [30], and cell cycle kinases [7,31] have been shown to force cancer cells into dormancy. On the other hand, dormancy-inducing molecules can be targeted. Various factors that promote dormancy have been identified. For example, recent research identified mitogen- and stress-activated kinase 1 (MSK1) as a positive regulator of metastatic dormancy in ER^+^ breast cancer by modulating transcriptional factors involved in luminal cell differentiation [32]. MSK1 downregulation is related to increased DTC bone homing and growth capacities [32]. Regucalcin (RGN) promotes prostate cancer dormancy by enhancing key hallmarks of tumor dormancy [33]. The lysophosphatidic acid receptor 1 (LPA1) inhibitor Debio-0719 (Table 1) induces breast cancer dormancy by reducing proliferative markers such as Ki67 and ERK, while increasing the growth inhibiting pathway p38 [34]. Sustained activation of the IRF7/INF-β/IFNAR pathway has been observed in ER^-^ dormant breast cancer cells that survived chemotherapy [35]. Conversely, the inactivation of this pathway disrupts cancer dormancy, leading to relapses and metastatic progression [35]. Ju et al. reported that COP9 signalosome 8 (CSN8) is essential for hypoxia-induced dormancy in colorectal cancer [27]. Upregulated CSN8 induces the expression of dormancy markers, such as p27 and DEC2, by activating the hypoxia inducible factor 1α (HIF-1α) signaling pathway [27].

The retinoic acid-regulated orphan nuclear receptor NR2F1 induces cell dormancy in head and neck squamous cell carcinoma [7], and its agonist C26 (Table 1) promotes DTC dormancy and inhibits lung metastasis [36]. As NR2F1 expression can be regulated by retinoic acid (RA) [37], all-trans retinoic acid (atRA) was tested, and the results suggest that atRA induces the expression of NR2F1 [7]. Furthermore, the DNA promoter methylation downregulated NR2F1 [7]. These findings prompted the idea of a combined treatment with the DNA-demethylating agent 5-azacytidine (AZA) and atRA. Cotreatment with AZA and atRA replicated NR2F1-dependent dormancy [7]. Currently, this combination is being tested clinically in prostate cancer patients experiencing relapse (NCT03572387).

Leukemia inhibitory factor (LIF) and its receptor LIFR provide a pro-dormancy signal to metastasized breast cancer cells in bone [38]. A humanized anti-LIF antibody, MSC-1, has been developed to inhibit LIF/LIFR signaling for treating patients with advanced solid tumors. The phase I clinical trial (NCT03490669) has been completed, demonstrating a strong safety profile for MSC-1, with MSC-1 also hacing showed an immunostimulatory function, supporting the use of MSC-1 with immune checkpoint inhibitors [39]. A follow-up phase II trial is currently underway to test the effect of MSC-1 in combination with a PD-L1 inhibitor, durvalumab, (NCT04999969) in patients with metastatic pancreatic ductal adenocarcinoma [40]. It is unclear whether MSC-1’s therapeutic effect is due to its pro-dormancy properties; however, MSC-1 is a promising agent that may be used to induce cell dormancy for cancer treatment.

### 3.2. Tumor Microenvironment-Related Dormancy

Supplying components of the dormant niche has also been shown to maintain dormancy in various settings. The pre-metastatic and metastatic niches are specialized microenvironments within distant organs that play a crucial role in the progression and dormancy of DTCs. These niches are constantly remodeled through extracellular matrix (ECM) changes and tumor-specific signals. Once DTCs reach these niches, the metastatic microenvironment further shapes their behavior, influencing whether proliferation or dormancy will occur [41]. In many cases, the microenvironment within the metastatic niche can induce dormancy, particularly through signals that suppress cell proliferation and promote survival in a dormant state. For example, microenvironmental cues such as TGFβ2 [15,21,42], BMP7 [42,43,44], and IFN-γ [45,46,47] have been shown to promote dormancy in DTCs.

Targeting the tumor microenvironment (TME) has emerged as an important strategy for regulating cancer cell dormancy. Cancer cells interact closely with the ECM, immune and non-immune cells, and blood vessels, which together constitute the TME. Hypoxia within the TME has been reported to induce dormancy in various cancer cells [6,24,25,26,27]. The ECM is intricately linked to all hallmarks of cancer, and many studies have highlighted its role in dormancy. For example, dormant breast cancer cells have been shown to remodel fibronectin to maintain dormancy through α5β1 integrin-mediated adhesion and Rho-associated kinase (ROCK)-mediated cell tension [21]. Similarly, dormant cells assemble type III collagen to sustain their dormant state, with type III collagen promoting dormancy by activating the discoidin domain receptor/signal transducer and activator of transcription 1 (DDR1/STAT1) signaling, thereby creating a type III collagen-enriched environment [20]. ECM components can induce dormancy in DTCs by binding to syndecan receptors on their surface [48]. Additionally, ER^+^ breast cancer cells have been observed to use fibronectin to increase their number of dormant cells in the presence of fibroblast growth factor 2 (FGF-2) [49]. These findings suggest that the interactions between DTCs and the ECM play a crucial role in determining whether DTCs remain dormant or proliferate, and these interactions may be targetable for therapeutic intervention.

Immune-related mechanisms can also maintain tumor cells in a dormant state, thus preventing their progression into larger lesions. In a B cell lymphoma mouse model, CD8^+^ T cells induced and maintained dormancy through INF-**γ** signaling [45]. Similarly, tumors derived from the 4T07 mouse breast cancer cell line exhibit a higher frequency of CD39^+^PD-1^+^CD8^+^ T cells compared to those derived from the 4T1 cell line. 4T07 tumors have been shown to prime CD8^+^ T cells to induce cell dormancy and inhibit metastasis [47].

### 3.3. Inhibition of Angiogenesis

Angiogenic therapeutics aimed at inducing cancer cell dormancy have been pursued since the 1970s. Tumor dormancy can be induced through preventing neovascularization, a concept first demonstrated in 1972 by Gimbrone Jr. et al., who showed that tumor fragments placed in nonvascularizable microenvironments remained dormant [50]. Angiogenesis inhibitors, such as angiostatin, as well as other therapeutics that target vascular endothelial growth factors (VEGFs), have been shown to induce dormancy in tumors by limiting blood supply and restricting tumor growth potential [51,52,53,54]. Further support of this strategy includes the fact that experimentally induced dormant tumors have significantly less angiogenic density [55].

### 3.4. Epigenetic Regulation of Dormancy

Dormant DTCs maintain their dormant state through epigenetic mechanisms, including histone modifications and heightened DNA methylation. These modifications lead to a more condensed chromatin structure and affect both transcriptional and post-transcriptional gene regulation [56]. For instance, the upregulation of the epigenetic gene Tet2 has been shown to induce cell dormancy by catalyzing hydroxymethylation to generate 5-hmC in cell cycle inhibitors [57]. Additionally, the overexpression of macroH2A variants from the H2A histone family induces a dormant phenotype in head and neck squamous cell carcinoma cells [58]. Several FDA-approved therapeutics targeting the epigenome are used to treat advanced and relapsed cancers. Histone deacetylase inhibitors (HDACis) are among these agents, with studies demonstrating their effects on cancer dormancy. For example, HDACis have been shown to epigenetically induce the leukemia inhibitory factor receptor (LIFR), which promotes dormancy in breast cancer cells [38], and activate a pro-dormancy program in breast cancer [59]. HDACis also induce growth arrest and dormancy in uveal melanoma cells [60].

Inducing a dormant state in cancer cells presents a promising therapeutic strategy in the fight against cancer by leveraging the natural ability of cancer cells to enter a dormant state, thereby preventing their proliferation and spread. By forcing cancer cells into dormancy, we can potentially reduce tumor growth, delay metastasis, and prolong patient survival without the severe side effects often associated with conventional treatments that aim to eliminate cancer cells outright. Furthermore, dormant cells, while not eradicated, may be kept in a controlled state, reducing the risk of recurrence and allowing for continuous monitoring and targeted intervention should reactivation occur. However, the challenge lies in maintaining dormancy indefinitely or transitioning it into a terminally dormant state, where reactivation is not a threat. Continued research into the molecular mechanisms and environmental cues that govern dormancy will be critical in developing therapies that effectively induce and sustain dormancy, offering a new paradigm in cancer treatment focused on long-term disease management and improved quality of life for patients.

## 4. Strategies of Eliminating Cancer Cell Dormancy

Dormant cells inherently resist traditional systemic therapies such as chemotherapy, which creates a significant barrier to preventing relapse. To address these cells, innovative strategies are needed, either to directly eliminate them, reactivate them for improved targeting with chemotherapy, or modify their microenvironment. This section explores these strategies and evaluates their effectiveness in both preclinical and clinical settings.

### 4.1. Direct Killing of Dormant Cancer Cells

Recent studies have demonstrated that dormant cancer cells can be selectively targeted by certain therapies. For instance, in a BRCA1/p53-deficient mouse model, non-proliferating platinum-chemotherapy resistant cancer cells were found to be sensitive to the alkylating agent nimustine, leading to complete eradication of tumors [61]. Mechanistically, DNA damage caused by nimustine is not repaired as efficiently as that caused by platinum-based chemotherapy drugs [61]. While platinum-induced intrastrand crosslinks can be removed during the G0-G1 phase of the cell cycle [62], nimustine induces interstrand crosslinks that impair DNA damage repair in dormant cells. Conversely, dormant myeloma cells were resistant to the alkylating agent melphalan but responsive to the proteasome inhibitor bortezomib [63]. The mechanisms underlying this difference remain unclear. These studies suggest that different cancer types respond variably to alkylating agents, highlighting the need for further investigation into the effects of these alkylating agents on various dormant cancer cells. Additionally, anticancer peptide-mimetic copolymers have been shown to damage cell membranes and kill dormant PC-3 cells resistant to docetaxel [64].

Inhibiting factors that promote dormancy can also lead to the elimination of dormant cells. The survival kinase DYRK1B (dual-specificity tyrosine phosphorylation-regulated kinase 1B) has been shown to be upregulated in dormant cancer cells, promoting dormancy by stabilizing the CDK inhibitor and inducing the degradation of cyclin D [65]. The knockdown of DYRK1B or the use of DYRK1B inhibitors led to the death of dormant cells and the induction of apoptosis in various cell lines [65]. Touny et al. reported that while inhibiting Src family kinase (SFK) signaling kept breast cancer cells in a dormant state, combining SFK inhibitors with MEK1/2 inhibitors (upstream activators of MAPK) killed dormant cells and reduced metastatic burden [30]. Additionally, inhibiting redox regulators such as NRF2 or DRP1 made dormant breast cells more vulnerable to cisplatin [66]. The regulator of G protein signaling 2 (RGS2) promotes dormancy and the survival of dormant cells in non-small-cell lung cancer, and its depletion led to apoptosis in these cells [67]. The protein kinase RNA-like ER kinase (PERK) also contributes to the survival of dormant cancer cells [31,68]. The inhibition of PERK with the potent and selective inhibitor HC-5404 (Table 1) eradicated dormant D-Hep3 cells as well as dormant disseminated cancer cells in the bone marrow [31]. This PERK inhibitor has recently completed its phase I clinical trial (NCT04834778), demonstrating a favorable safety profile and efficacy in patients with advanced solid tumors [69].

Several studies have reported that autophagy induces and maintains dormancy in cancer cells, while the inhibition of autophagy lead to the reactivation of dormant cells [70,71,72]. The autophagy inhibitor hydroxychloroquine selectively reduces the viability of dormant cells while leaving proliferative cells unaffected [70]. Mechanistically, autophagy inhibition in dormant cells impairs the clearance of damaged mitochondria and by-products, such as reactive oxygen species (ROS) [70]. Additionally, the autophagy regulator mTORC1 promotes the survival of dormant cancer cells [73], suggesting that combining mTOR and autophagy inhibitors could be a more effective strategy [74,75]. These findings have prompted several ongoing phase II clinical trials evaluating the efficacy of FDA-approved autophagy inhibitors, such as hydroxychloroquine, either alone or in combination with other drugs, to prevent relapses in breast cancer patients with DTCs in their bone marrow. For example, the CLEVER trial (NCT03032406) investigated hydroxychloroquine in combination with the mTOR inhibitor everolimus to treat breast cancer patients during remission stages to prevent recurrence [76]. The results indicated that hydroxychloroquine and everolimus effectively reduced DTCs, and the clearance of DTCs was related to improved outcomes [77]. Two additional clinical trials are currently ongoing to confirm and extend the results of the CLEVER trial: the ABBY trial (NCT04523857) and the PALAVY trial (NCT04841148).

The ABBY trial is examining the effects of hydroxychloroquine in combination with the CDK4/6 inhibitor abemaciclib to determine whether targeting DTCs in the bone marrow can reduce DTC numbers and potentially eradicate them. Additionally, the PALAVY trial is exploring the safety and early efficacy of the checkpoint inhibitor avelumab or hydroxychloroquine, with or without the CDK4/6 inhibitor palbociclib, in ER^+^ breast cancer patients with DTCs in the bone marrow. This trial is based on the premise that autophagy is activated in dormant cells as a survival mechanism, and inhibiting this process may lead to the elimination of dormant cells [74]. Taken together, inhibitors of autophagy hold promise as adjuvant treatments for eradicating dormant cancer cells and preventing cancer recurrence.

### 4.2. Reactivating Dormant Cancer Cells

Reactivating dormant cells to induce their re-entry into the cell cycle can make them more susceptible to conventional anti-proliferative therapeutics, offering a potential strategy for eradicating dormant tumor cells and addressing cancer cell dormancy. Many studies have suggested combining this reactivation strategy with conventional chemotherapy. However, given that cancer is inherently characterized by unchecked growth, the deliberate promotion of cell division must be carefully evaluated. Proper management is essential, as reactivation could potentially exacerbate disease progression if not carefully controlled.

Protein phosphatase 2A (PP2A) inhibitors, such as LB1 (Table 1), can reawaken dormant cancer cells by stimulating them to enter mitosis. This is achieved through the activation of AKT and interference with p53-mediated cell cycle arrest, thereby enhancing the cells’ sensitivity to chemotherapy. This approach has demonstrated efficacy in treating head and neck squamous cell carcinoma [78], glioblastoma multiforme, and neuroblastoma [79]. Additionally, dormancy-inducing agents that enhance the expression of SMAD4 have been found to maintain disseminated cancer cells in a dormant state. However, the loss of TGF-β-SMAD4 signaling can facilitate the proliferation of these cells [80], suggesting that targeting SMAD4 might effectively reactivate dormant cells and induce their re-entry into the cell cycle. Jiang et al. developed a peptide–drug conjugate, DTX-P7, which combines heptapeptide P7 with docetaxel. The peptide P7 specifically binds to the cell surface heat shock protein Hsp90 in non-small-cell lung cancer, enabling targeted delivery. This conjugate was found to awaken dormant A549/CD133^+^ cells by degrading a negative regulator of cell cycle progression, thus promoting their re-entry into the cell cycle and subsequent cell death [81]. Furthermore, the Fbxw7 gene is highly expressed in dormant breast cancer cells, and its deletion has been shown to reactivate dormant cells. Combining Fbxw7 ablation with chemotherapy reduced the number of DTCs, even when applied after tumor cell dissemination [82].

Breast cancer DTCs have been reported to exhibit epithelial–mesenchymal plasticity. Dormant DTCs typically display a mesenchymal state, which is mediated by the transcriptional factor ZFP281 [83]. The reacquisition of an epithelial state, driven by the overexpression of E-cadherin, can trigger the reawakening of these cells [84]. Targeting E-cadherin may affect the dormancy–proliferation switch in breast cancer DTCs, potentially forcing dormant cells to re-enter the cell cycle.

### 4.3. Targeting the Dormant Cell Microenvironment

Upon reaching their metastatic site, DTCs encounter either favorable or hostile conditions, depending on the specific microenvironment of the site, which determines their dormant or proliferative phenotype. Factors in the TME that influence the fate of DTCs include neighboring cells, angiogenesis, inflammation, and the ECM.

**Figure 1:**
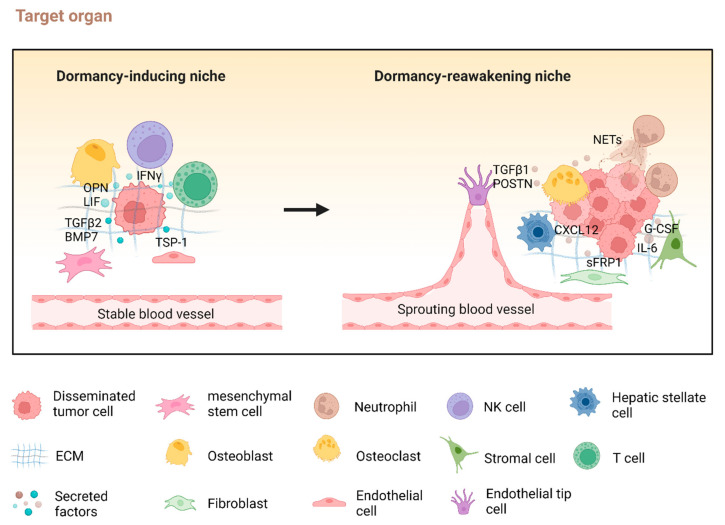
Interactions between disseminated tumor cells (DTCs), adjacent cells, and the tumor microenvironment modulate cellular dormancy. Disseminated tumor cells (DTCs) interact with components of the tumor microenvironment, modulating the dormancy-inducing and dormancy-reawakening niche. Created with BioRender.com.

Neighboring cells at metastatic sites regulate the dormant or proliferative states of DTCs by interacting with them and secreting cytokines and environmental cues (Figure 1) [75]. For example, in the bone marrow, myeloma cells can remain dormant by interacting with osteoblasts in the endosteal niche [63], while osteoclast-mediated remodeling of this niche can reactivate dormant myeloma cells [63]. Osteoblast-induced dormancy has also been observed in prostate cancer cells, with the focal adhesion kinase (FAK) inhibitor PF-562271 (Table 1) effectively mimicking this osteoblast-induced dormancy [85]. Similarly, an osteoclastic niche promoted by VCAM-1 helps breast cancer cell escape dormancy and activate micrometastasis [86]. In the bone marrow niche, NG2^+^/Nestin^+^ mesenchymal stem cells (MSCs) promote breast cancer DTC dormancy through TGFβ2 and BMP7, and depleting MSCs or the knockout of TGFβ2 in MSCs reactivates dormant cells [42]. In head and neck squamous cell carcinoma models, DTCs tend to stay dormant at bone marrow metastatic sites but are proliferative at lung metastatic sites [15]. Mechanistically, increased TGFβ2/MAPK signaling in the bone marrow induces dormancy, while lower levels of TGFβ2 in the lungs facilitate the reawakening of dormant DTCs [15]. This suggests that local expression levels of TGFβ2/MAPK are crucial in determining whether DTCs exhibit a dormant or proliferative phenotype. The role of TGFβ in regulating dormancy is further supported by Gao et al., who found that the secreted TGF-β ligand Coco promotes the reactivation of dormant breast cancer cells in the lung by inhibiting BMP signaling [87]. Notably, Coco’s effect on tumor dormancy is specific to the lung and does not apply to bone or brain tissues [87], suggesting that the regulation of dormant-cell reactivation is organ-specific, depending on the local microenvironment.

In breast cancer models, docetaxel has been shown to awaken dormant cancer cells by injuring stromal cells, leading to the release of IL-6 and granulocyte colony-stimulating factor (G-CSF). This cytokine release activates MEK/ERK signaling, resulting in the reactivation of dormant cells [88]. The role of IL-6 in dormancy reactivation is further supported by evidence from DTC samples in the bone marrow of breast cancer patients [89] and CD133^+^ stem-like cells [90]. Additionally, supplying growth factors such as insulin-like growth factor 1 (IGF1) and G-CSF has been shown to affect dormant cells. In leukemic cells, G-CSF administration increases the number of proliferating cells while reducing dormant cells in the bone marrow [91]. Combining G-CSF with traditional chemotherapy effectively drives dormant leukemia-initiating cells into the cell cycle, making them more sensitive to treatment and facilitating their eradication [91]. Similarly, supplementation with IGF1 stimulates the proliferation of dormant breast tumor cells, leading to increased DTC outgrowth and higher Ki67^+^ cell counts [92]. Conversely, the dual inhibition of IGF1/IGF1 receptor (IGF-1R) and AKT with linsitinib effectively eliminates dormant cancer cells that lack the expression of mutant KRAS or c-MYC in pancreatic cancer models [93].

Specific spatial organizations at the metastatic site influence whether DTCs remain dormant or enter a proliferative state. For example, dormant breast cancer cells are found in the perisinusoidal regions of the bone marrow, while proliferative cells are located in the nonsinusoidal regions [94]. The microvascular niche surrounding DTCs also affects their fate: DTCs near sprouting vessels tend to proliferate, whereas those near stable microvascular endothelium remain dormant [95]. Mechanistically, thrombospondin-1 (TSP-1) derived from endothelial cells induces dormancy, whereas TGFβ1 and periostin (POSTN) derived from sprouting tip cells accelerate cell growth (Figure 1) [95].

In breast cancer, biomechanical forces induced by the ECM promote tumor cell dormancy through DDR2 (discoidin domain receptor 2)/STAT1 signaling. Conversely, the degradation of ECM components triggers the re-entry of dormant cells into proliferation [96]. Similarly, in lymphoblastic leukemia, the ECM molecule osteopontin (OPN), secreted by osteoblasts, helps anchor blast cells in a dormancy-supporting microenvironment within the bone marrow. Pretreatment with OPN neutralization has been shown to reactivate dormant cells in leukemia mouse models [97]. Additionally, the remodeling of the ECM protein laminin induces the reawakening of dormant cancer cells by activating integrin α3β1 signaling [98].

Lung inflammation induces the formation of neutrophil extracellular traps (NETs), which concentrate neutrophil elastase and matrix metalloproteinase 9 (MMP9) at laminin. This leads to laminin’s proteolytic remodeling and the subsequent reawakening of dormant cancer cells [98]. Consistent with this, a mouse model of liver ischemia–reperfusion injury demonstrated that surgical stress triggered the awakening of DTCs through NET formation [99]. Further studies support these findings, showing that LPS-induced inflammation in the lung [100], neutrophil release of proinflammatory proteins [101], and surgery-related inflammation in both the lung [102] and liver [103] can also reactivate dormant cancer cells.

Several studies have highlighted that aging and fibrosis within the microenvironment can facilitate the reawakening of dormant cancer cells [5,104,105,106,107]. For example, fibrilla fibronectin has been shown to induce dormancy awakening in HEp3 cells by inhibiting the cell cycle repressor p38 and de-repressing ERK1/2 [29]. In dormant breast cancer cells, the production of ECM components such as type I collagen and fibronectin trigger a transition from dormancy to proliferation. This transition is regulated through integrin β1 activation, leading to the phosphorylation of ERK-dependent myosin light chain (MLC) by MLC kinase (MLCK) [5,106]. The role of integrin β1 in tumor dormancy reactivation is further supported by findings that integrin β1-deficient tumors exhibit prolonged dormancy [108]. Additionally, the upregulation of platelet-derived growth factor (PDGF)-C mediates the reawakening and proliferation of dormant DTCs in ER^+^ breast cancer within an aged and fibrotic microenvironment, leading to metastatic outgrowth [105]. Similarly, aged lung fibroblasts have been shown to promote the reawakening of dormant melanoma cells in the lung through increased secretion of the WNT antagonist sFRP1 and decreased expression of WNT5A [104]. Furthermore, in mouse models, natural killer (NK) cells maintain breast cancer dormancy in the liver through IFNγ-induced dormancy. Activated hepatic stellate cells (aHSCs), which drive fibrosis, can disrupt NK cell-mediated cancer dormancy and trigger an exit from dormancy. Mechanistically, aHSCs facilitate this process by secreting CXCL12, which inhibits NK cell proliferation [46].

The primary tumor microenvironment plays a crucial role in programming DTCs for a dormant phenotype at secondary sites. For instance, Borriello et al. reported that DTCs acquire the dormancy marker NR2F1 through interactions with macrophages within the primary tumor’s microenvironment. The depletion of macrophages reduced DTC survival after extravasation at secondary sites, while interactions with macrophages before reaching these secondary sites increased their retention and survival [109]. These findings suggest that interactions with macrophages in the primary tumor microenvironment are essential for the dissemination and dormancy of tumor cells.

These studies suggest that DTCs are in constant communication with their surrounding microenvironment. The interactions between residual DTCs and their microenvironment are critical in determining whether they adopt a dormant or proliferative phenotype (Figure 1). Generally, metastatic sites with a stable, homeostatic environment may induce and maintain DTC dormancy. Conversely, changes such as angiogenesis, inflammation, or ECM remodeling that disrupt this homeostatic balance can trigger the awakening of DTCs from dormancy.

### 4.4. Immunotherapeutic Approaches

Immune cells are a major component of the tumor microenvironment, and recent studies have increasingly focused on their role in regulating cancer cell dormancy. Dormant cancer cells are known to evade immunosurveillance through various mechanisms. For instance, these cells often downregulate NK cell activators, thereby evading NK cell detection and clearance while maintaining a stem-like state that enables their colonization [110]. Similarly, dormant cells may downregulate the expression of major histocompatibility complex I (MHC I) to escape recognition by CD8^+^ T cells [111,112]. Additionally, dormant cancer cells can create an immune-suppressive environment characterized by reduced numbers of infiltrating immune cells and dysfunctional T cells [12]. Targeting the key molecules involved in these immune evasion mechanisms presents promising therapeutic opportunities.

In myeloid leukemia, dormant leukemia cells are resistant to cytotoxic T cell (CTL)-mediated killing due to the upregulation of the checkpoint protein PD-L1 and B7.1 [113]. However, these resistant cells can be targeted by NK cells derived from mice vaccinated with DA1-3b cells transduced with CXCL10, demonstrating a promising strategy for overcoming immune evasion [114]. Furthermore, treatment with anti-PD-1 drugs has been shown to reduce the number of dormant DTCs in the lungs of mice [47]. These studies suggest that immune checkpoint inhibitors, such as PD-1/PD-L1 inhibitors, may be effective in eradicating dormant cancer cells and preventing metastatic relapses. This approach is currently being evaluated in an ongoing phase II clinical trial examing breast cancer patients with DTCs in the bone marrow (NCT04841148).

To counteract these immune evasion strategies, enhancing the interaction between dormant DTCs and tumor antigen-specific T cells is crucial. This can be achieved through several strategies aimed at increasing the number of antigen-specific T cells capable of recognizing and eliminating dormant cells. These strategies include the use of T cell-based vaccines, the adoptive transfer of engineered T cell receptors, and chimeric antigen receptor T (CAR-T) cells [112]. Such approaches aim to boost the immune system’s ability to target and eradicate dormant tumor cells, thereby improving the efficacy of immunotherapeutic interventions.

## 5. Conclusions and Perspectives

Cancer cell dormancy is an adaptive survival strategy employed in hostile environments and represents a crucial stage in cancer development. The accurate detection and quantification of dormant cancer cells in patients constitutes a great challenge in translating dormancy-targeting therapies into clinical practice [115]. A promising clinical trial (NCT 02732171) is currently focused on screening DTCs using bone marrow aspirate from patients within 5 years of completing breast cancer therapy [116]. Another clinical trial suggested that the status of DTCs can help identify high-risk patients after chemotherapy (NCT00248703) [117]. Additionally, studies have shown promising results for treating DTCs in the bone marrow of breast cancer patients using zoledronic acid (NCT00172068) [118,119].

Understanding the mechanisms that maintain dormancy, the factors that reawaken dormant cells (Table 2), the microenvironment surrounding primary and secondary sites, and the role of immunosurveillance against dormant cells is fundamental for developing effective therapies targeting dormancy. Dormant cancer cells are resistant to cell death and refractory to therapeutic drugs, contributing to cancer recurrence and metastasis. Effective cancer treatment must either sustain the dormant state or effectively eliminate dormant cells. However, both approaches have limitations: sustaining dormancy requires lifelong treatment, which is impractical, while attempting to kill dormant cells by inducing their reactivation may lead to worse patient outcomes if the therapies fail. To address these challenges, a deeper understanding of the underlying mechanisms is essential.

## Figures and Tables

**Table 1:** Chemical structures of dormancy-specific therapeutic agents.

Compound	Function	Target	Structure
PF-562271	Inducing dormancy	FAK	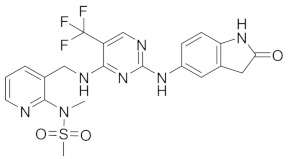
Debio-0719	Inducing dormancy	LPA1	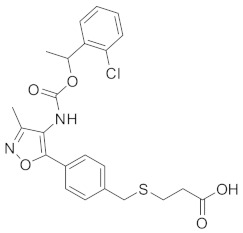
C26	Inducing dormancy	NR2F1	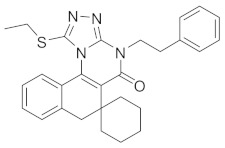
HC-5404	Eradicating dormancy	PERK	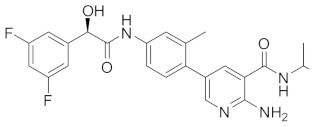
LB1	Reawakening dormancy	PP2A	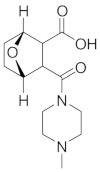

**Table 2:** Summary of factors that induce and reawaken dormant cancer cells.

	Factor	Cancer Type	Ref.
Dormancy-inducing factors	TGFβ2	Breast cancer, head and neck squamous cell carcinoma	[15,21,42]
	IFN-γ	Breast cancer, lymphoma	[45,46,47]
	IFN-β	Breast cancer	[35]
	TNFα	Breast cancer	[47]
	WNT5a	Prostate cancer, melanoma	[104,120]
	BMP7	Breast cancer, prostate cancer	[42,43,44]
	TSP-1	Breast cancer	[95]
	LIF	Breast cancer	[38]
	Hypoxia	Colorectal cancer, lung cancer, breast cancer, head and neck squamous cell carcinoma	[6,24,26,27]
	HDACis	Breast cancer	[59,60]
	STAT1	Squamous cell carcinoma, breast cancer	[20,96]
	NR2F1	Head and neck squamous cell carcinoma	[7,36]
	SMAD4	Head and neck squamous cell carcinoma	[80]
	Autophagy	Breast cancer, ovarian cancer	[70,71,72]
	Mesenchymal state	Breast cancer, colorectal cancer	[27,83,84]
Reawakening factors	TGFβ1	Breast cancer	[95]
	POSTN	Breast cancer	[95]
	integrin β1	Lung cancer, breast cancer	[5,28,98,106,108]
	VCAM-1	Breast cancer	[86]
	E-cad	Breast cancer	[84]
	NF-kB	Breast cancer	[86]
	MMP-9	Breast cancer	[98]
	Coco	Breast cancer	[87]
	LB1	Head and neck squamous cell carcinoma, glioblastoma multiforme, neuroblastoma	[78,79]
	Inflammation and NETs	Lung cancer	[98,99,100,101,102,103]
	Aging	Melanoma, breast cancer	[104,105]
	Fibrosis	Breast cancer, squamous cell carcinoma	[5,29,105,106]
	Epithelial state	Breast cancer	[83,84]
	IL-6	Breast cancer	[88,89,90]
	G-CSF	Breast cancer, leukemia	[88,91]
	sFRP1	Melanoma	[104]

## Data Availability

No new data were created or analyzed in this study.
